# Psychometric Properties of the Persian Version of the Delirium Care Self‐Efficacy Scale for Intensive Care Unit Nurses: A Methodological Study

**DOI:** 10.1002/nop2.70800

**Published:** 2026-09-24

**Authors:** Hossein Fadaei, Mehraban Shahmari, Reza Nemati‐Vakilabad, Fateme Ebrahimi Belil

**Affiliations:** ^1^ Department of Medical‐Surgical Nursing, School of Nursing and Midwifery Ardabil University of Medical Sciences Ardabil Iran; ^2^ Department of Emergency Nursing, School of Nursing and Midwifery Ardabil University of Medical Sciences Ardabil Iran

**Keywords:** delirium, intensive care units, nurse, psychometrics, self‐efficacy

## Abstract

**Aim(s):**

Currently, no valid and reliable instrument exists in Persian to assess intensive care unit (ICU) nurses' self‐efficacy in delirium care, which limits the ability to identify educational needs and evaluate targeted interventions in Iranian critical care settings. This study aimed to evaluate the psychometric properties of the Persian version of the Delirium Care Self‐Efficacy Scale (P‐DCSE‐I) for ICU nurses in Iran.

**Design:**

A methodological study with a cross‐sectional design.

**Methods:**

The original scale was translated into Persian using a forward‐backward method. Psychometric properties were assessed among 209 ICU nurses in Ardabil, Iran. Face and content validity were evaluated qualitatively and quantitatively. Construct validity was examined through Exploratory Factor Analysis (EFA) and Confirmatory Factor Analysis (CFA). Reliability was assessed using internal consistency and test–retest reliability.

**Results:**

Qualitative face and content validity were confirmed following minor revisions. The Kaiser‐Meyer‐Olkin (KMO) measure was 0.921, and Bartlett's Test of Sphericity was significant (*χ* ^2^(78) = 1286.45, *p* < 0.001), confirming the adequacy of the data for factor analysis. EFA revealed a clear two‐factor structure (“Confidence in delirium assessment,” with 7 items, and “Confidence in delirium management,” with 6 items), explaining 73.6% of the total variance. CFA confirmed the model with excellent goodness‐of‐fit indices (*χ* ^2^/df = 2.341, CFI = 0.948, RMSEA = 0.047). The internal consistency was acceptable for the total scale (*α* = 0.86) and subscales (*α* = 0.82–0.85). The test–retest reliability was also excellent (ICC = 0.84).

**Conclusion:**

The P‐DCSE‐I demonstrates strong validity and reliability. It is a suitable instrument for measuring delirium care self‐efficacy among ICU nurses in Iran and is useful for clinical assessment and research.

**Implications for the Profession and/or Patient Care:**

This valid and reliable instrument enables the assessment of ICU nurses' confidence in delirium care, facilitating the identification of educational needs and the evaluation of targeted training interventions to improve patient safety and quality of care for critically ill patients with delirium.

**Impact:**

- Currently, no valid and reliable instrument exists in Persian to assess ICU nurses' self‐efficacy in delirium care, limiting the ability to identify educational needs and evaluate interventions in Iranian critical care settings.
- The Persian version of the DCSE‐I demonstrated strong psychometric properties, including a two‐factor structure, acceptable internal consistency, and acceptable test–retest reliability.
- This instrument will support ICU nurses, nurse educators, and researchers in Iran and other Persian‐speaking populations by facilitating evidence‐based assessment and improvement of delirium care practices.

**Reporting Method:**

This study adhered to the Consensus‐based Standards for the Selection of Health Measurement Instruments (COSMIN) checklist guidelines for study design and reporting.

**Patient or Public Contribution:**

A total of 209 ICU nurses participated in this study. No patients, service users, caregivers, or members of the public were involved in the design, conduct, analysis, interpretation, or preparation of this manuscript.

## 1 Introduction

Delirium, characterized by an acute and fluctuating disturbance in consciousness and cognition, is one of the most prevalent and serious complications in intensive care units (ICUs) (Jeong and Cho 2023). Its incidence is alarmingly high, impacting up to 80% of mechanically ventilated patients and a substantial number of all critically ill individuals (Wu et al. 2023). A recent systematic review and meta‐analysis conducted in Iran reported an overall delirium prevalence of 22% among hospitalized patients, with rates reaching 20.2% in cardiac surgery patients and 44.3% among the elderly population (Erfani et al. 2025). Furthermore, a cross‐sectional study in Iranian ICUs found that 22.43% of patients developed delirium during their admission, with moderate and severe thirst increasing the risk of delirium by approximately 4 and 14 times, respectively (Barani et al. 2025). The repercussions of ICU delirium are significant and extend well beyond the immediate hospital stay. It is independently linked to prolonged mechanical ventilation, longer ICU and hospital stays, increased healthcare costs, long‐term cognitive deficits, and higher mortality rates (Fiest et al. 2021; Jeong and Cho 2023). Given the substantial clinical and economic burden of delirium in Iranian ICUs, prioritizing its prevention, early identification, and management is crucial for delivering high‐quality, patient‐centered critical care. Therefore, adopting targeted interventions to mitigate this condition is not just advantageous but imperative for improving patient outcomes within the critical care environment (Imai et al. 2025; Lobo‐Valbuena et al. 2021).

Acknowledging the crucial role of nurses in this area, international guidelines strongly advocate for routine delirium monitoring utilizing validated assessment tools, such as the Confusion Assessment Method for the ICU (CAM‐ICU) (Dos Santos et al. 2022) or the Intensive Care Delirium Screening Checklist (ICDSC) (Diao et al. 2024). ICU nurses, as frontline caregivers, possess both continuous presence and clinical expertise, enabling them to detect subtle changes in a patient's mental status. The unique contribution of ICU nursing in the context of delirium lies in 24‐h bedside presence, which positions nurses to identify the acute and fluctuating onset of delirium symptoms that may be missed during periodic physician rounds (Kazemi et al. 2025). ICU patients differ markedly from other medical populations due to the high prevalence of mechanical ventilation (affecting up to 75% of patients), deep sedation, and the complex interplay of multiple delirium risk factors including metabolic disturbances, polypharmacy, and severe underlying illness (van den Boogaard et al. 2024). These factors make delirium detection particularly challenging, as symptoms may be masked by sedation or attributed to the critical illness itself (Chang et al. 2023). Accurately identifying delirium and administering appropriate non‐pharmacological and pharmacological interventions is vital to alleviating its adverse effects (Hebeshy et al. 2024; Lange et al. 2023).

Despite the availability of validated delirium assessment tools in Iran, including the Persian version of the CAM‐ICU (Mohammad et al. 2019), evidence suggests that ICU nurses face persistent challenges in delirium recognition. A recent Iranian study found that 81% of ICU nurses had poor perception of delirium assessment, 79.9% never performed delirium screening, and 93.1% had not participated in delirium training courses (Kazemi et al. 2025). This gap between guideline recommendations and clinical practice underscores the need to understand the factors influencing nurses' confidence and competence in delirium care. Moreover, although screening tools are available, no instrument measures nurses' perceived self‐efficacy—their belief in their ability to perform essential tasks—a critical determinant of clinical behaviour and performance (Chang et al. 2023).

Successfully integrating delirium care practices into daily nursing routines is a complex challenge and is often executed suboptimally (Nie et al. 2024; Yu et al. 2023). Extensive research indicates that barriers to effective delirium management are multifaceted and interrelated. Key contributors include overwhelming workloads that leave nurses stretched thin, a perceived lack of time to conduct thorough assessments, and insufficient knowledge of delirium recognition and management strategies (Bramley et al. 2021; Jeong and Cho 2023). Studies from the Middle East region have similarly documented low levels of delirium knowledge among ICU nurses, with 83% of nurses in Iraq having not participated in any delirium‐related training (Najafi Ghezeljeh et al. 2023). A particularly critical factor is nurses' lack of confidence in their ability to perform necessary assessments and interventions effectively. Bandura's social cognitive theory defines this perception of one's capability to execute essential tasks as self‐efficacy (Nickerson 2024). Within the nursing profession, self‐efficacy emerges as a powerful predictor of clinical performance and behaviour; studies indicate that nurses with higher levels of self‐efficacy are significantly more likely to engage in complex clinical behaviours, persist in overcoming challenges, and ultimately achieve better patient outcomes (Dong et al. 2025; Nie et al. 2024). Therefore, enhancing self‐efficacy through targeted training and support may be vital in improving the overall effectiveness of delirium care in clinical settings.

A critical evaluation of existing instruments underscores the need for a validated scale specifically designed to assess delirium care self‐efficacy. While tools such as the Generalized Self‐Efficacy Scale (GSES) (Schwarzer and Jerusalem 1995) and the Nursing Professional Self‐Efficacy Scale (NPSES) (Caruso et al. 2016) are widely used, they suffer from significant limitations. The GSES addresses self‐efficacy in a general context and does not specifically target the unique challenges of delirium management, making it less applicable to ICU nurses facing this critical issue. Similarly, the NPSES, while focusing on essential critical care skills, does not encompass the nuanced aspects of delirium care, especially the psychological and emotional dimensions of self‐efficacy that nurses face in high‐pressure environments. Bandura argued that self‐efficacy is domain‐specific, meaning its assessment content must vary across different clinical fields (Chang et al. 2023). Therefore, a general self‐efficacy measure cannot adequately capture nurses' confidence in the specific skills required for delirium assessment and management. The lack of such a targeted instrument in Iran limits the ability to identify educational needs and evaluate intervention effectiveness.

In contrast, the DCSE‐I, developed by Chang et al. (2023), provides a tailored assessment that directly measures self‐efficacy related to delirium management, including the attitudes, skills, and behaviours crucial for delivering effective care. The scale has been validated in Taiwan and, more recently, in mainland China, demonstrating strong psychometric properties with a two‐factor structure and high internal consistency. This focused approach not only enhances the scale's relevance for ICU nurses but also supports targeted educational interventions, which can improve patient outcomes and increase caregivers' confidence in managing delirium.

The need to conduct this study in the Iranian context is multifaceted. First, while the impact of delirium on ICU patient outcomes is a global issue, the effective implementation of delirium care practices is heavily influenced by localized factors. Currently, approximately 73% of Iranian ICU nurses have not completed a formal delirium education course, and 69.4% express willingness to use assessment tools despite low rates of actual implementation (Kazemi et al. 2025). These factors include tailored nursing curricula, interdisciplinary team dynamics, resource availability, and cultural perceptions of critical care within the healthcare system. The substantial gaps in knowledge and practice observed among Iranian ICU nurses, combined with the high delirium prevalence in Iranian hospitals, highlight the urgent need for a valid instrument to assess self‐efficacy—a key predictor of clinical performance—to inform targeted educational interventions and quality improvement initiatives (Erfani et al. 2025). Directly translating a tool without thorough validation within the distinct socio‐cultural and professional environment of Iranian ICUs risks rendering it irrelevant and ineffective in accurately measuring the intended construct.

Secondly, there is a notable lack of a culturally and linguistically adapted instrument to assess delirium care self‐efficacy among Iranian ICU nurses. As a result, nursing administrators and educators in Iran lack a valid and reliable instrument to identify specific gaps in nurses' confidence, evaluate the effectiveness of targeted training programs, and ultimately shape policies to strengthen the clinical workforce. This study addresses this gap by rigorously translating, culturally adapting, and psychometrically validating the Persian version of the Delirium Care Self‐Efficacy Scale for ICU nurses (P‐DCSE‐I) in Iran.

## 2 Methods

### 2.1 Design

This study employed a methodological research design with a cross‐sectional component. Methodological studies are specifically conducted to develop, evaluate, or validate instruments and measurement tools, making this design the most appropriate choice for assessing the psychometric properties of the P‐DCSE‐I. The cross‐sectional component was incorporated to collect data from a diverse sample of ICU nurses at a single point in time, which is consistent with standard practices in instrument validation research. This design is particularly suitable because psychometric evaluation requires adequate sample sizes to perform robust statistical analyses such as Exploratory Factor Analysis (EFA) and Confirmatory Factor Analysis (CFA), and a cross‐sectional approach allows for efficient data collection while minimizing participant burden. Additionally, cross‐sectional designs are widely used in methodological studies to establish preliminary evidence of reliability and validity before conducting more resource‐intensive longitudinal investigations. The study was conducted from February to August 2025 in Ardabil, Iran, following the Consensus‐based Standards for the Selection of Health Measurement Instruments (COSMIN) checklist guidelines for study design and reporting.

### 2.2 Setting and Sample

The study was conducted across four educational‐therapeutic centers in Ardabil, Iran: Imam Khomeini Hospital (internal medicine, cardiology, and nephrology), Fatemi Hospital (trauma and emergency center), Alavi Hospital (neurology and obstetrics/gynaecology), and Imam Reza (urology). These tertiary referral centers, affiliated with Ardabil University of Medical Sciences, provide specialized care for critically ill patients from both urban and rural areas across Ardabil province, with ICU bed occupancy rates approaching 100%. The participating hospitals include general teaching hospitals with specialized ICUs (medical‐surgical, trauma, cardiology, and neurology), each with 6–12 active beds, a nurse‐to‐patient ratio of approximately 1:2, and standard critical care monitoring systems.

The target population comprised all nurses working in the ICUs of the four participating hospitals. A census sampling method was used, in which all eligible ICU nurses were invited to participate. The recruitment process involved: obtaining a comprehensive list of ICU nurses from each hospital's nursing office; screening potential participants against inclusion criteria (registered nurses with at least a bachelor's degree and a minimum of 6 months of ICU experience); nurses on extended leave were excluded; approaching eligible nurses individually, explaining the study purpose, and providing written information sheets; obtaining written informed consent; and distributing self‐administered questionnaires, collected in sealed opaque envelopes to maintain anonymity. Questionnaires with more than 20% missing data were excluded.

The sample size was determined by evaluating the requirements for both Exploratory Factor Analysis (EFA) and Confirmatory Factor Analysis (CFA), with CFA as the primary factor influencing the final sample size due to its more rigorous demands for parameter estimation. For EFA, several methodological references suggest a minimum participant‐to‐item ratio of 5 to 10, with 10 participants per item regarded as optimal (Kline and Santor 1999; Tinsley and Tinsley 1987). Since the DCSE‐I scale consists of 13 items, this implies a minimum requirement of 130 participants. However, relying solely on this guideline is insufficient for final sample size determination, as it neglects important factors such as communality levels, factor loadings, the number of factors, and overall model complexity.

CFA, which has stricter statistical requirements, typically recommends a sample size of at least 150 to 200 participants to ensure stable parameter estimates and adequate statistical power. Wolf et al. (2013) illustrated through Monte Carlo simulations that sample size requirements for structural equation modelling can vary from 30 to 460 cases, depending on model characteristics, including the number of indicators and factors, the magnitude of factor loadings and path coefficients, and the presence of missing data. Importantly, MacCallum et al. (1999) noted that the required sample size is influenced by the level of variable communality and the degree of factor overdetermination. When communalities are consistently high (greater than 0.6), the impact of sample size is less critical; however, moderate communalities necessitate larger samples. Furthermore, Comrey and Lee (2013) proposed a general scale for assessing sample size in factor analysis: 50 = very poor, 100 = poor, 200 = fair, 300 = good, 500 = very good, 1000 or more = excellent. Hair et al. (2019) provided specific guidelines indicating that for standardized factor loadings of 0.40 or higher, a sample size of 200 is adequate; for loadings of 0.45 or higher, 150 participants are sufficient; and for loadings of 0.50 or higher, a sample size of 120 is adequate.

The present study employed both EFA and CFA, with the more stringent CFA requirements determining the final target sample size. Based on several considerations, a sample of 200 participants was deemed adequate. The scale used in the study comprises 13 items divided into 2 factors, reflecting a relatively straightforward and well‐defined structure. The participant‐to‐item ratio, approximately 15:1 (209 participants/13 items), exceeds the recommended minimum of 10:1. According to MacCallum et al. (1999), a sample size of 100–200 is sufficient when communalities are moderate to high. Given that the final factor loadings ranged from 0.62 to 0.91, indicating moderate to high communalities, the sample of 209 participants is justified. Hair et al. (2019) indicated that for factor loadings of 0.40 or higher, a sample size of 200 is adequate. Since the present study's loadings (0.62–0.91) significantly exceed this threshold, the sample size is appropriate.

In this study, 230 ICU nurses were invited to participate. Out of these, 209 completed and analysable questionnaires were received. The difference between invited and analysed participants is due to 21 exclusions: 8 cases were removed for incomplete responses (over 20% missing data), 7 participants withdrew consent during the survey, and 6 did not return the questionnaire. Consequently, the final sample of 209 reflects the number of complete responses after exclusions, rather than just the initial target. The response rate of 90.9% (209 out of 230) and the final sample size of 209 exceed the minimum requirement of 200 participants set by the CFA‐driven sample size criteria (Myers et al. 2011; Tabachnick and Fidell 2013).

A significant methodological consideration is that both EFA and CFA were conducted using the same group of 209 participants. While traditional guidelines recommend splitting the sample into two independent subsamples for EFA and CFA to achieve genuine cross‐validation, this approach involves considerable practical and methodological challenges (Fernández‐Hernández et al. 2022; Zhou 2022). Randomly splitting a sample of 209 participants would create two subsamples of approximately 104–105 participants each, which falls below the recommended minimum of 150–200 participants needed for stable CFA parameter estimates. Furthermore, recent methodological research shows that split‐sample cross‐validation does not enhance generalizability and can introduce variability based on random group selection (Fletcher 2023). Fernández‐Hernández et al. argued that criticisms of conducting EFA and CFA on the same dataset also apply to split‐sample validation, as randomly partitioned groups tend to be statistically identical, with differences arising solely from randomization; true independent samples are essential for actual cross‐validation (Fernández‐Hernández et al. 2022). Given the practical limitations of recruiting ICU nurses from a small target population and the acknowledged drawbacks of split‐sample methods, we used the full sample of 209 participants for both EFA and CFA. This maximized statistical power and the stability of parameter estimates. Using the full sample allows for more stable estimates and greater precision than split‐sample approaches, particularly given the available sample size for this study. This approach aligns with the viewpoint that when additional recruitment for a separate sample is not feasible, leveraging the full sample instead of a split sample provides more reliable estimates, especially with smaller sample sizes.

### 2.3 Participant Information Form

The research team developed a two‐part participant information form to collect demographic and professional data. Part 1 included demographic characteristics (age, gender, educational level, marital status, and employment status). Part 2 included professional characteristics (total nursing experience, ICU experience, completion of delirium education courses, and willingness to use delirium assessment tools). The form was developed based on a literature review (Mohammad et al. 2019; van den Boogaard et al. 2024) and reviewed by an expert panel of ten nursing faculty members.

### 2.4 Delirium Care Self‐Efficacy Scale for ICU Nurses (DCSE‐I)

The Delirium Care Self‐Efficacy Scale for ICU nurses (DCSE‐I) was developed by Chang et al. (2023) to measure ICU nurses' confidence in providing delirium care (Chang et al. 2023). The scale consists of 13 items across two factors: “Confidence in delirium assessment” (7 items: 1, 2, 3, 4, 5, 6, 7) and “Confidence in delirium management” (6 items: 8, 9, 10, 11, 12, 13). Responses are recorded on a 5‐point Likert scale, ranging from 1 (very unsure) to 5 (completely confident). The total score is the sum of all item scores, ranging from 13 to 65, with a higher score indicating greater self‐efficacy. The minimum possible score is 13, and the maximum possible score is 65. No predetermined cut‐off point exists for categorizing participants into “high” or “low” self‐efficacy groups, as the scale was designed to measure continuous levels of self‐efficacy rather than classify individuals. This approach is consistent with the original development and validation study, which also treated the scale as a continuous measure without establishing categorical cut‐offs. The original scale's content validity index (CVI) was measured at 0.98, while the overall Cronbach's α was found to be 0.94, with subscale values between 0.86 and 0.92.

### 2.5 Pilot Study

Before the main data collection, a pilot study was conducted to assess the clarity, comprehensibility, and feasibility of the translated P‐DCSE‐I and the Participant Information Form. The pilot study was carried out with a convenience sample of 20 ICU nurses from Imam Khomeini Hospital and Fatemi Hospital who met the same inclusion criteria as the main study participants. These nurses were not included in the main study sample to avoid contamination of the psychometric evaluation. The purpose of the pilot study was threefold: (1) to evaluate the clarity and wording of the translated items, (2) to estimate the time required to complete the questionnaire, and (3) to identify any potential difficulties with the questionnaire format or instructions. Participants completed the questionnaire and provided feedback on any ambiguous, difficult, or unclear items. The average completion time was approximately 5–10 min, and participants identified no significant issues with item comprehension, formatting, or instructions. Minor wording adjustments were made based on participant feedback to enhance clarity. The pilot study confirmed the feasibility and acceptability of the questionnaire for use in the main study, and no modifications to the scale structure or scoring were required.

### 2.6 Translation Procedure

The translation of the DCSE‐I from English into Persian followed a standard forward‐backward translation procedure in accordance with the established cross‐cultural adaptation guideline by Beaton et al. (2000) to ensure conceptual, semantic, and idiomatic equivalence. The process involved the following stages:

- Forward translation: Two bilingual translators whose native language was Persian independently translated the original English version of the DCSE‐I into Persian. One translator was a professional with a nursing background who was familiar with the study's concepts, while the other was a professional translator unfamiliar with the research objectives. This approach yielded two Persian versions (T1 and T2).
- Synthesis of translations: The two translators and the research team compared T1 and T2 to resolve any discrepancies. Through discussion, a single reconciled Persian forward‐translation version (T‐12) was produced.
- Backward translation: Two other bilingual translators independently back‐translated the synthesized Persian version (T‐12) into English, blinded to the original scale. This step was crucial for identifying any misunderstandings or conceptual deviations that may have occurred during the forward translation
- Expert committee review: A panel of experts, comprising the initial translators, the back‐translators, a methodologist, and five nursing faculty members, was convened. The committee compared the original DCSE‐I with the back‐translated versions and the reconciled Persian version. Their goal was to review all reports and reach a consensus on any semantic, idiomatic, experiential, or conceptual inconsistencies. The final Persian version was established after consensus.

### 2.7 Psychometric Evaluation

#### 2.7.1 Face Validity

The face validity of the P‐DCSE‐I was evaluated through a two‐tiered qualitative and quantitative methodology. In the qualitative phase, 10 ICU nurses were purposefully selected to assess the clarity, relevance, and appropriateness of each item in relation to the intended construct. Participants offered critical insights on the wording, comprehensibility, and structural coherence of the items, resulting in minor adjustments to enhance precision and ensure cultural relevance.

For the quantitative evaluation, the Impact Score (IS) (IS = Frequency (%) × Importance) was computed to ascertain the perceived relevance of individual items. ICU nurses rated the Importance of each statement using a 5‐point Likert scale (1 = not important to 5 = extremely important). Items with an IS of 1.5 or higher were retained, validating their appropriateness for inclusion in the final scale (Bornstein 1996; Johnson 2021).

#### 2.7.2 Content Validity

To assess the qualitative content validity of the P‐DCSE‐I, an independent expert panel of 10 specialists with expertise in intensive care nursing, nursing education, instrument development, and clinical psychology reviewed the scale. This panel was distinct from the expert committee involved in the translation process to ensure the independence and objectivity of the content‐validation procedure. The experts evaluated each item for grammar, wording, item allocation, and scaling, as well as its relevance and clarity in measuring delirium care self‐efficacy among ICU nurses in the Iranian context. Based on the panel's feedback, we made minor phrasing modifications to several items to improve comprehension and cultural appropriateness.

We evaluated quantitative content validity by calculating the Content Validity Index (CVI) and Content Validity Ratio (CVR). The same panel of 10 experts rated each item on a 4‐point scale for relevance, clarity, and necessity. The Item‐CVI (I‐CVI) was calculated as the proportion of experts who rated each item 3 or 4, with all items achieving an I‐CVI above the acceptable threshold of 0.78. The CVR was assessed based on experts' ratings of each item's essentiality, using Lawshe's table with a minimum acceptable value of 0.62 for a panel of 10 experts (Almanasreh et al. 2019; Lawshe 1975).

#### 2.7.3 Construct Validity

##### 2.7.3.1 Exploratory Factor Analysis (EFA)

Before conducting EFA, we performed item discrimination analysis to evaluate each item's ability to distinguish between nurses with higher and lower levels of delirium care self‐efficacy. Item discrimination refers to the degree to which an item can differentiate individuals with high levels of the target construct from those with low levels. For this purpose, we calculated the corrected item‐total correlation (CITC) for each of the 13 items. The CITC represents the Pearson correlation between each item and the scale's total score excluding that item, with values above 0.30 generally considered acceptable. Items with CITC values below 0.20 are typically considered to have poor discrimination and may be candidates for revision or removal.

EFA was employed to investigate the underlying factor structure of the P‐DCSE‐I. We first assessed the data's suitability for EFA using the following criteria: a Kaiser‐Meyer‐Olkin (KMO) measure greater than 0.80 was considered excellent for factor analysis, and a statistically significant Bartlett's Test of Sphericity (*p* < 0.05) was required to proceed (Pett et al. 2003). Factor extraction was performed using the Maximum Likelihood method. We determined the number of factors retained using multiple criteria, including visual inspection of the scree plot and eigenvalues greater than 1.0 (Kaiser's criterion). To achieve a simple, interpretable factor structure, we applied an oblique rotation method (Promax). The following cut‐off points were used for the interpretation of factor loadings: factor loadings greater than 0.50 were considered significant, loadings between 0.30 and 0.50 were considered moderate, and loadings below 0.30 were considered weak. Items with cross‐loadings exceeding 0.32 on multiple factors were flagged for further examination. The analysis aimed to identify a factor solution that accounted for a substantial proportion of the total variance and was clearly interpretable (Widaman and Helm 2023).

##### 2.7.3.2 Confirmatory Factor Analysis (CFA)

To verify the factor structure identified through the EFA, Confirmatory Factor Analysis (CFA) was conducted. The hypothesized two‐factor model, comprising “Confidence in delirium assessment” and “Confidence in delirium management,” was tested. We used the maximum likelihood method to estimate the model parameters. The model fit was evaluated using a comprehensive set of goodness‐of‐fit indices with the following pre‐defined criteria for an acceptable model: Chi‐square/degrees of freedom (*χ* ^2^/df) < 3, Root Mean Square Error of Approximation (RMSEA) < 0.08, Standardized Root Mean Square Residual (SRMR) < 0.08, Goodness‐of‐Fit Index (GFI) > 0.90, Comparative Fit Index (CFI) > 0.90, Tucker‐Lewis Index (TLI) > 0.90, and Relative Fit Index (RFI) > 0.90. We also examined the parsimony‐adjusted indices, Parsimonious Comparative Fit Index (PCFI) and Parsimonious Goodness‐of‐Fit Index (PGFI), with values greater than 0.50 indicating acceptable model parsimony. We assessed the significance of the factor loadings using critical ratios (*T*‐values), with values greater than 1.96 indicating statistical significance at the *p* < 0.05 level (Hox 2021; Sathyanarayana and Mohanasundaram 2024; West et al. 2023).

##### 2.7.3.3 Convergent and Discriminant Validity

To further assess the construct validity of the Persian version of the Delirium Care Self‐Efficacy Scale, we examined both convergent and discriminant validity using the Fornell‐Larcker criterion (Fornell and Larcker 1981). Convergent validity was evaluated using two indicators: Composite Reliability (CR) and Average Variance Extracted (AVE). Composite Reliability values greater than 0.7 were considered acceptable, indicating adequate internal consistency. Average Variance Extracted values greater than 0.5 were considered satisfactory, demonstrating that the latent construct explains more than half of the variance in its indicators.

Discriminant validity was assessed by comparing the square root of the AVE for each factor with the correlations between factors. According to the Fornell‐Larcker criterion, discriminant validity is established when the square root of the AVE for each construct is greater than its correlation with other constructs. Additionally, we examined the Maximum Shared Variance (MSV), which should be less than the AVE for each factor to provide further evidence of discriminant validity.

#### 2.7.4 Reliability

We evaluated the reliability of the P‐DCSE‐I by assessing both its internal consistency and stability over time. Internal consistency, which reflects the extent to which items within the scale measure the same construct, was assessed using three complementary indices: Cronbach's alpha (*α*), McDonald's omega ($\omega_{t}$), and Maximal Reliability (MaxR(H)). Values greater than 0.70 generally indicate acceptable internal consistency for research purposes (Kalkbrenner 2023; Malkewitz et al. 2023; McNeish 2018).

We assessed scale‐score stability by administering the same scale to participants on two separate occasions. We used the Intraclass Correlation Coefficient (ICC) to measure this. Specifically, we employed a two‐way random‐effects model with an absolute agreement definition for our analysis. A subset of the sample, consisting of 40 participants, completed the scale again after a 2‐week interval. Typically, ICC values greater than 0.75 are interpreted as indicating excellent reliability (Xue et al. 2021).

#### 2.7.5 Data Analysis

Data analysis was performed using IBM SPSS Statistics for Windows, version 24 (IBM Corp., Armonk, NY, USA), and IBM SPSS AMOS (Version 24). Before the primary analysis, we screened the data for missing values, outliers, and adherence to multivariate analysis assumptions. We assessed the normality of each item's distribution using both graphical methods (Q–Q plots) and statistical tests (Kolmogorov–Smirnov and Shapiro–Wilk tests).

Given that the data were collected using a 5‐point Likert scale, which produces ordinal‐level responses, we carefully considered the appropriate estimation method for CFA. Although Likert‐scale data are technically ordinal, methodological literature widely supports using Maximum Likelihood Estimation (MLE) under specific conditions. We justify our use of MLE based on the following evidence‐based reasons: First, extensive simulation studies have demonstrated that MLE is remarkably robust to violations of multivariate normality when certain conditions are met, particularly when the number of response categories is at least five, and the sample size is adequate (≥ 200) (Li 2016; Rhemtulla et al. 2012). With five response categories and a sample of 209 participants, our data satisfy these conditions, making MLE a defensible and reliable choice. Second, when ordinal data exhibit skewness or kurtosis beyond acceptable limits, alternative estimators such as Weighted Least Squares Mean‐ and Variance‐adjusted (WLSMV) are often recommended. However, in our dataset, the skewness and kurtosis values for all items were within the acceptable range of |2| and |7|, respectively, as recommended by Curran et al. (1996). This indicates that our data, while ordinal, approximate a continuous distribution sufficiently well to permit the use of MLE without substantial bias. Third, MLE provides distinct practical advantages, including the ability to compute a full range of fit indices and employ robust standard errors. To further improve the accuracy of our estimates and account for residual non‐normality, we applied the Bollen‐Stine bootstrap correction with 2000 bootstrap samples, a method designed to adjust *p*‐values and standard errors when using MLE with non‐normal or ordinal data (Bollen and Stine 1992). This bootstrap approach is widely accepted as an effective strategy to mitigate the limitations of MLE with ordinal data. Fourth, recent methodological guidelines (Mindrila 2010; Wang and Wang 2019) suggest that when the number of categories is five or more, and the sample size exceeds 200, the differences between MLE and robust ordinal estimators (such as WLSMV) are negligible in terms of parameter estimation, factor loadings, and model fit interpretation. Given our sample characteristics, we are confident that MLE with bootstrap correction provides accurate and unbiased results. Finally, we examined multivariate outliers using Mahalanobis distance, and cases with a significant value (*p* < 0.001) were reviewed and retained if no data entry error was found, as they were considered valid representations of the population (McNeish and Wolf 2023). To evaluate multivariate normality, Mardia's test of multivariate skewness and kurtosis was performed. Given the ordinal nature of the Likert‐scale data, a violation of multivariate normality was anticipated. A two‐tailed *p*‐value of less than 0.05 was considered statistically significant for all analyses.

#### 2.7.6 Common Method Bias Assessment

Given that all data were collected through self‐reported questionnaires, the potential for Common Method Bias (CMB) was a concern. To minimize this risk, we implemented several procedural remedies during data collection, as recommended by Podsakoff et al. (2003). These included: (a) ensuring respondent anonymity to reduce social desirability bias, (b) using clear and concise item wording to prevent ambiguity, (c) separating the measurement of the predictor and criterion variables where possible, and (d) assuring participants that there were no right or wrong answers.

To statistically assess the presence of CMB, two complementary approaches were employed. First, Harman's Single‐Factor Test was conducted using Exploratory Factor Analysis, where all 13 items were forced into a single unrotated factor. If a single factor accounts for more than 50% of the total variance, CMB is considered to be present (Podsakoff et al. 2003). Second, the Common Latent Factor (CLF) method was applied within the CFA framework. In this approach, a common method factor was added to the hypothesized two‐factor model, with all items loading onto this additional factor. The model fit was then compared with the baseline model. If adding the common method factor does not substantially improve model fit, it suggests that CMB is not a significant threat (Podsakoff et al. 2003; Williams et al. 1989).

## 3 Results

### 3.1 Characteristics of the Participants

The study included 209 ICU nurses. The majority were female (89.9%), held a Bachelor's degree (88.9%), and were married (60.3%). The mean age of the participants was 33.84 (SD = 6.99) years, with a mean total work experience of 12.84 (SD = 8.61) years and 6.39 years (SD = 5.55) of experience specifically in the ICU. Notably, most nurses (73.2%) had not completed a formal delirium education course; however, a majority (69.4%) expressed willingness to use delirium assessment tools (Table 1).

**TABLE 1:** Socio‐demographic characteristics of participants (*n* = 209).

Characteristic	Category	Mean ± SD	
Age (years)		33.84 ± 6.99	
Total working experience (years)		12.84 ± 8.61	
Working experience in the ICU (years)		6.39 ± 5.55	
		**N**	**%**
Gender	Male	21	10.1
	Female	188	89.9
Education level	Bachelor's degree	186	88.9
	Master's degree	23	11.1
Marital status	Single	75	35.9
	Married	126	60.3
	Divorce/widow	8	3.8
Completion of the delirium education course	Yes	56	26.8
	No	153	73.2
Willingness to use delirium assessment tools	Yes	145	69.4
	No	64	30.6

### 3.2 Assessment of Multivariate Normality

Mardia's test of multivariate normality revealed a significant departure from multivariate normality (Multivariate Skewness: *β* = 12.847, *p* < 0.001; Multivariate Kurtosis: *κ* = 58.231, *p* < 0.001). This finding is consistent with the ordinal nature of the Likert‐scale data and justifies using the Bollen‐Stine bootstrap correction in our CFA analyses.

### 3.3 Face Validity

During the qualitative phase, participants assessed the clarity, relevance, and cultural appropriateness of the translated items. Feedback revealed that the items effectively conveyed concepts pertinent to delirium care self‐efficacy within the ICU context. However, participants recommended minor linguistic modifications to improve the naturalness of the Persian phrasing. The Impact Score was determined for the quantitative evaluation, yielding scores between 2.5 and 3.8. All items surpassed this threshold, validating their appropriateness for the intended construct. Consequently, no items were discarded during this phase (Table 2).

**TABLE 2:** The results for the face and content validity of the P‐DCSE‐I (*n* = 10).

Item	Impact score	I‐CVI	CVR
*Factor 1: Confidence in delirium assessment*			
1. Can tell the difference between hypoactive delirium and depression	2.5	1.0	1.0
2. I can evaluate if the patient exhibits signs of attention disorder	2.7	0.9	0.8
3. I can distinguish if the patient has been overdosed with a sedative	3.8	0.9	0.8
4. I can evaluate if the patient exhibits signs of confusion	2.5	1.0	1.0
5. I can evaluate if the patient exhibits signs of disorientation	3.2	0.9	0.8
6. I can differentiate the hypoactive symptoms of delirium from the patient	3.7	1.0	1.0
7. I am aware of the timing for performing a consciousness evaluation on a patient under sedation	3.2	0.9	0.8
*Factor 2: Confidence in delirium management*			
8. I can provide a sense of orientation to the patient during my nursing rotation	2.5	1.0	1.0
9. I am confident in easing the patient's discomfort	3.4	1.0	0.8
10. I feel confident I try my best to keep the patient in a comfortable and quiet environment	2.7	0.9	0.8
11. I can discuss with the physician about how to improve the sleeping quality for patients by using drugs	2.5	1.0	0.8
12. I can explain the nursing procedures to the patients before performing them	2.5	1.0	0.8
13. I can actively search for information about providing care for a patient with delirium	3.4	0.8	0.8

*Note:* Impact Score ≥ 1.5, CVI ≥ 0.79, and CVR ≥ 0.62 were considered acceptable.

Abbreviations: CVR, Content Validity Ratio; I‐CVI, Item‐Content Validity Index.

### 3.4 Content Validity

During the qualitative evaluation, a panel of experts thoroughly examined each item for appropriateness, clarity, and relevance to self‐efficacy in delirium care within the ICU setting. Their detailed feedback prompted minor but significant wording changes to enhance clarity and ensure each item conveyed its intended meaning while preserving the original constructs. Ultimately, every item was unanimously deemed conceptually relevant to the target domain, affirming its importance in assessing and improving self‐efficacy in delirium management among ICU nurses.

Quantitative content validity was evaluated using the I‐CVI and CVR. I‐CVI values for individual items ranged from 0.8 to 1.0, with all 13 items exceeding the acceptable threshold of 0.78. The CVR values ranged from 0.8 to 1.0, with all items exceeding the minimum acceptable value of 0.62 for a panel of 10 experts. These findings indicate that experts unanimously considered all items as essential and pertinent, thereby affirming satisfactory quantitative content validity for the entire instrument (Table 2).

### 3.5 Construct Validity

The item discrimination analysis showed that all 13 items demonstrated acceptable to excellent discrimination, with corrected item‐total correlations ranging from 0.52 to 0.79. These values exceeded the recommended threshold of 0.30, confirming that all items effectively differentiate between nurses with higher and lower levels of delirium care self‐efficacy. This analysis identified no items for removal.

The KMO measure was calculated at 0.921, indicating sampling adequacy. At the same time, Bartlett's Test of Sphericity was significant (*χ* ^2^(78) = 1286.45, *p* < 0.001), demonstrating that the correlations among items were sufficiently strong for EFA. Utilizing Maximum Likelihood extraction and Promax rotation, the analysis uncovered a clear two‐factor structure. All 13 items exhibited significant factor loadings (greater than 0.715) on their respective factors, with no cross‐loadings exceeding 0.32. Factor 1, labelled “Confidence in delirium assessment,” accounted for 41.7% of the variance, whereas Factor 2, titled “Confidence in delirium management,” contributed 31.9%. Together, these two factors explained a cumulative total of 73.6% of the variance. The communalities for all items were above 0.50, confirming that the factor solution represented each item effectively. Detailed results are available in Table 3.

**TABLE 3:** Results of the exploratory factor analysis for the P‐DCSE‐I (*n* = 209).

Item	Factor loading		Communalities
	Factor 1	Factor 2	
1. Can tell the difference between hypoactive delirium and depression	0.812	0.166	0.685
2. I can evaluate if the patient exhibits signs of attention disorder	0.861	0.105	0.785
3. I can distinguish if the patient has been overdosed with a sedative	0.776	0.231	0.677
4. I can evaluate if the patient exhibits signs of confusion	0.715	0.221	0.643
5. I can evaluate if the patient exhibits signs of disorientation	0.920	0.082	0.712
6. I can differentiate the hypoactive symptoms of delirium from the patient	0.885	0.011	0.641
7. I am aware of the timing for performing a consciousness evaluation on a patient under sedation	0.784	0.172	0.513
8. I can provide a sense of orientation to the patient during my nursing rotation	0.084	0.863	0.684
9. I am confident in easing the patient's discomfort.	0.133	0.895	0.627
10. I feel confident I try my best to keep the patient in a comfortable and quiet environment	0.145	0.886	0.669
11. I can discuss with the physician about how to improve the sleeping quality for patients by using drugs	0.091	0.907	0.690
12. I can explain the nursing procedures to the patients before performing them.	0.232	0.828	0.651
13. I can actively search for information about providing care for a patient with delirium	0.097	0.786	0.652
Eigenvalue	5.630	4.978	
% of Variance	41.7%	31.9%	
Cumulative %	41.7%	73.6%	

*Note:* Extraction method: Maximum likelihood; Rotation method: Promax (Oblique) with Kaiser normalization.

**FIGURE 1:**
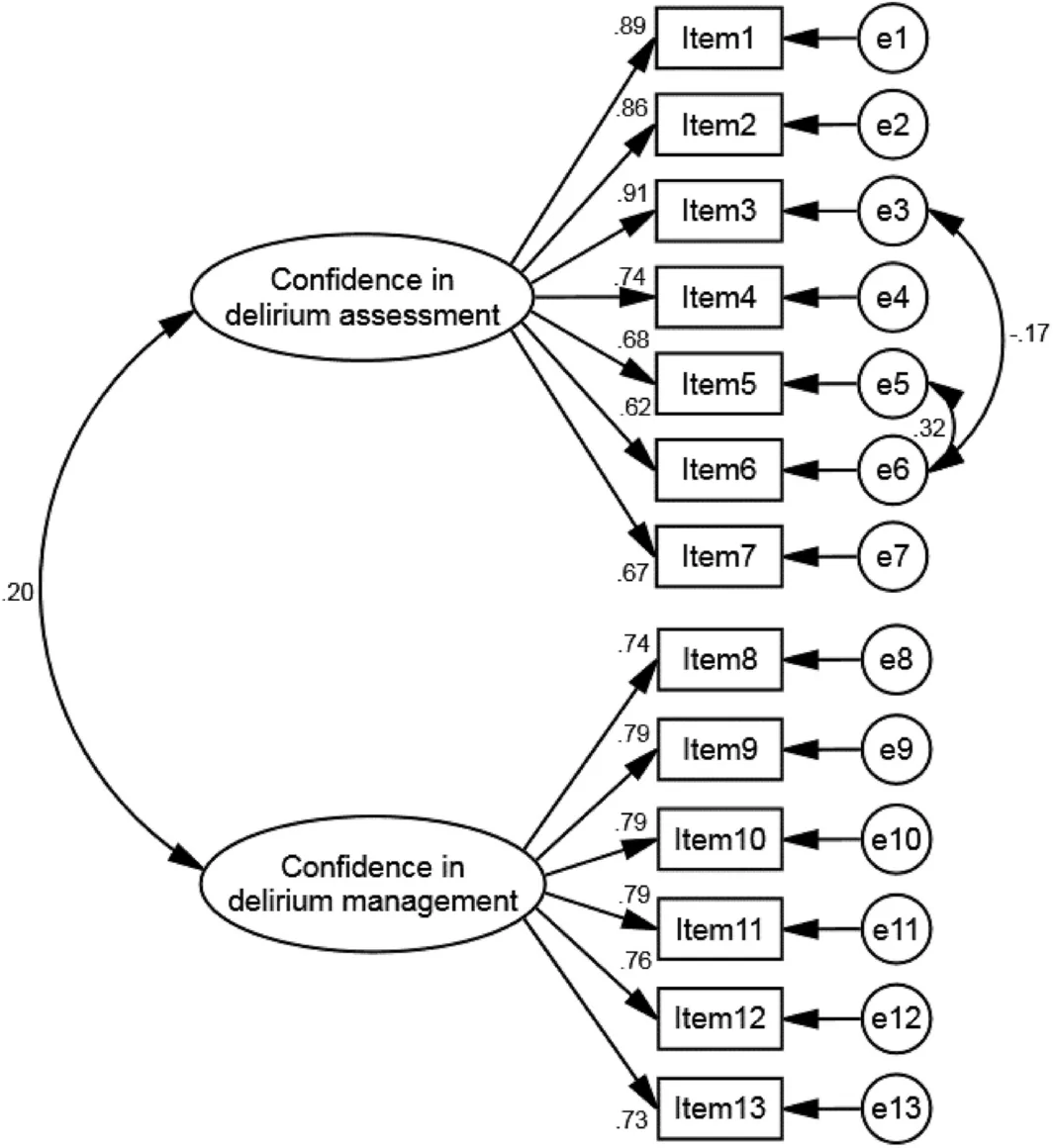
Confirmatory factor analysis model with standardized estimates (*n* = 209).

We tested the hypothesized two‐factor model using CFA. The results demonstrated a good fit of the model to the data, as all goodness‐of‐fit indices met the predefined criteria for an acceptable model. The chi‐square statistic was *χ* ^2^(64) = 145.142 (*p* < 0.001); however, given the sensitivity of the chi‐square test to sample size, additional fit indices were examined, which all indicated acceptable model fit: *χ* ^2^/df = 2.341, RMSEA = 0.047, SRMR = 0.033, GFI = 0.921, CFI = 0.948, TLI = 0.934, RFI = 0.920, PCFI = 0.753, and PGFI = 0.631 (Table 4). The standardized factor loadings for all items were statistically significant, with *T*‐values ranging from 9.54 to 31.38 (all *T*‐values > 1.96, *p* < 0.001). The loadings ranged from 0.62 to 0.91 (Figure 1), indicating a strong relationship between each item and its respective latent factor (Table 5).

**TABLE 4:** Goodness of fit statistics for CFA models of the P‐DCSE‐I (*n* = 209).

Fit index	Cutoff criteria for good fit	Estimate	Bootstrap 95% CI (Lower CL–Upper CL)	Bootstrap *p*‐value
Chi‐Square (*χ* ^2^)	*p*‐value > 0.05	*χ* ^2^(64) = 145.142	(131.52–159.84)	0.068
*p*‐value for *χ* ^2^	> 0.05 (Acceptable)	< 0.001	—	—
*χ* ^2^/df	< 3.0 (Acceptable)	2.341	(2.11–2.58)	—
RMSEA	< 0.08 (Acceptable)	0.047	(0.039–0.056)	0.712
SRMR	< 0.08 (Acceptable)	0.033	(0.028–0.039)	—
GFI	> 0.90 (Acceptable)	0.921	(0.904–0.938)	—
CFI	> 0.90 (Acceptable)	0.948	(0.931–0.962)	—
TLI	> 0.90 (Acceptable)	0.934	(0.915–0.951)	—
RFI	> 0.90 (Acceptable)	0.920	(0.899–0.939)	—
PCFI	> 0.50 (Acceptable)	0.753	—	—
PGFI	> 0.50 (Acceptable)	0.631	—	—

*Note:* *χ* ^2^ *p*‐value: *p* > 0.05 indicates acceptable model fit (non‐significant chi‐square suggests good fit); Bootstrap *p*‐value: *p* > 0.05 indicates acceptable model fit after correction for non‐normality (Bollen‐Stine bootstrap). Bootstrap 95% CI values are presented as (Lower CL–Upper CL) for each fit index where applicable. “—” indicates that the value was not applicable or could not be estimated for that specific fit index.

Abbreviations: *χ* ^2^, Chi‐Square; *χ* ^2^/df, Chi‐Square/Degrees of Freedom; CFI, Comparative Fit Index; CI, Confidence Interval; GFI, Goodness‐of‐Fit Index; Lower CL, Lower Confidence Limit; PCFI, Parsimony Comparative Fit Index; PGFI, Parsimony Goodness‐of‐Fit Index; RFI, Relative Fit Index; RMSEA, Root Mean Square Error of Approximation; SRMR, Standardized Root Mean Square Residual; TLI, Tucker‐Lewis Index; Upper CL, Upper Confidence Limit.

**TABLE 5:** Standardized factor loadings, standard errors, and 95% confidence intervals for the CFA model (*n* = 209).

Item	Standardized loading	SE	*T*‐value	95% CI	*p*‐value
*Factor 1: Confidence in delirium assessment*					
1. Can tell the difference between hypoactive delirium and depression	0.89	0.033	26.97	(0.825–0.955)	< 0.001
2. I can evaluate if the patient exhibits signs of attention disorder	0.86	0.037	23.24	(0.787–0.933)	< 0.001
3. I can distinguish if the patient has been overdosed with a sedative	0.91	0.029	31.38	(0.853–0.967)	< 0.001
4. I can evaluate if the patient exhibits signs of confusion	0.74	0.054	13.70	(0.634–0.846)	< 0.001
5. I can evaluate if the patient exhibits signs of disorientation	0.68	0.060	11.33	(0.562–0.798)	< 0.001
6. I can differentiate the hypoactive symptoms of delirium from the patient	0.62	0.065	9.54	(0.493–0.747)	< 0.001
7. I am aware of the timing for performing a consciousness evaluation on a patient under sedation	0.67	0.061	10.98	(0.550–0.790)	< 0.001
*Factor 2: Confidence in delirium management*					
8. I can provide a sense of orientation to the patient during my nursing rotation	0.74	0.054	13.70	(0.634–0.846)	< 0.001
9. I am confident in easing the patient's discomfort	0.79	0.048	16.46	(0.696–0.884)	< 0.001
10. I feel confident I try my best to keep the patient in a comfortable and quiet environment.	0.79	0.048	16.46	(0.696–0.884)	< 0.001
11. I can discuss with the physician about how to improve the sleeping quality for patients by using drugs	0.79	0.048	16.46	(0.696–0.884)	< 0.001
12. I can explain the nursing procedures to the patients before performing them	0.76	0.051	14.90	(0.660–0.860)	< 0.001
13. I can actively search for information about providing care for a patient with delirium	0.73	0.055	13.27	(0.622–0.838)	< 0.001

*Note:* All factor loadings were statistically significant at *p* < 0.001. Significance criteria: *T*‐values > 1.96 were considered statistically significant (*p* < 0.05); 95% CIs that do not contain zero indicate statistical significance; *p*‐values < 0.05 were considered statistically significant.

Abbreviations: CFA, Confirmatory Factor Analysis; CI, Confidence Interval; SE, Standard Error; *T*‐value, Critical Ratio.

### 3.6 Common Method Bias Assessment

Harman's Single‐Factor Test revealed that the single unrotated factor accounted for only 34.8% of the total variance, well below the 50% threshold. This suggests that no single factor explains the majority of the variance, indicating that CMB is not a significant concern.

Additionally, the CLF method was applied within the CFA framework. A common method factor was added to the hypothesized two‐factor model, with all 13 items loading onto this additional factor. The results showed that adding the common method factor did not substantially improve model fit (ΔCFI = 0.008, ΔTLI = 0.006, ΔRMSEA = 0.004), and the common method factor explained minimal variance (2.9%). These findings further confirm that CMB does not pose a significant threat to the validity of our findings.

### 3.7 Convergent and Discriminant Validity

Convergent validity was established, as the CR values for both factors were above 0.70 (0.911 and 0.895), and the AVE for each factor exceeded the threshold of 0.50 (0.601 and 0.588). Furthermore, discriminant validity was confirmed, as the MSV for each factor (0.04) was less than its corresponding AVE value. This indicates that each factor shares more variance with its own indicators than with the other factor in the model (Table 5).

### 3.8 Reliability

The reliability of the P‐DCSE‐I was evaluated through internal consistency and test–retest reliability. The internal consistency was acceptable, as indicated by high values for McDonald's Omega ($\omega_{t}$), Cronbach's Alpha (*α*), and Maximal Reliability (MaxR(H)). For the ‘Confidence in delirium assessment’ subscale, the values were *α* = 0.82, *ω* = 0.82, and MaxR(H) = 0.82. The ‘Confidence in delirium management’ subscale showed similarly high reliability (*α* = 0.84, *ω* = 0.85, and MaxR(H) = 0.85). The overall scale also demonstrated strong internal consistency (*α* = 0.86, *ω* = 0.87, and MaxR(H) = 0.87). Furthermore, test–retest reliability over a 2‐week interval was acceptable, as indicated by ICC values of 0.84 for the first subscale, 0.86 for the second subscale, and 0.84 for the total scale, confirming the instrument's stability over time (Table 6).

**TABLE 6:** Results of the convergent, discriminant validity, and reliability of the P‐DCSE‐I (*n* = 209).

Factor	CR	AVE	MSV	*α*	$\omega_{t}$	MaxR(H)	ICC (95% CI: LCL—UCL)
Confidence in delirium assessment	0.91	0.60	0.04	0.82	0.82	0.827	0.84 (0.803–0.868)
Confidence in delirium management	0.89	0.58	0.04	0.84	0.85	0.852	0.86 (0.832–0.871)
Total (P‐DCSE‐I)				0.86	0.87	0.871	0.84 (0.801–0.869)

*Note:* Values in parentheses represent the 95% Confidence Interval (LCL–UCL). All ICC values were significant *p* < 0.001. Significance criteria: ICC values with 95% CIs that do not contain zero and *p*‐values < 0.05 were considered statistically significant.

Abbreviations: $\omega_{t}$, McDonald's omega coefficient; AVE, Average Variance Extracted; CI, Confidence Interval; CR, Composite Reliability; ICC, Intraclass Correlation Coefficient; LCL, Lower Control Limit; MaxR(H), Maximum Reliability (H); MSV, Maximum Shared Squared Variance; UCL, Upper Control Limit; *α*, Cronbach's alpha.

## 4 Discussion

The primary aim of the present study was to evaluate the psychometric properties of the Persian version of the Delirium Care Self‐Efficacy Scale (P‐DCSE‐I) for use among ICU nurses in Iran. Given the high prevalence of delirium in critically ill patients and the pivotal role nurses play in its prevention, recognition, and management, a valid and reliable tool to measure their perceived self‐efficacy is crucial. This study sought to determine the validity (face, content, and construct) and reliability (internal consistency and test–retest) of the translated scale. Beyond merely reporting these psychometric indices, this discussion critically interprets their meaning in relation to existing theoretical frameworks and prior empirical work, clarifies the novel contribution of the Persian version, and considers the cultural‐contextual factors that may influence its application.

In terms of face validity, our process mirrored the original study's approach by conducting qualitative pilot testing with the target population (ICU nurses). As in the original scale (Chang et al. 2023), participants in our study found the items clear and relevant, leading only to minor linguistic revisions to improve natural phrasing in Persian. This aligns with the core purpose of face validity: to ensure the instrument is understandable and appears relevant to the end user. The quantitative assessment in our study, using an Impact Score, provided an additional layer of objective measurement not explicitly detailed in the original publication and confirmed that all items were well received.

Regarding content validity, our results showed strong item‐level content validity indices (I‐CVI range: 0.8–1.0), nearly identical to the high I‐CVI range (0.800–1.000) reported for the Chinese version (Nie and Jiang 2025). The scale‐level content validity index for our study (implied by the high I‐CVIs) is comparable to the excellent S‐CVI/Ave of 0.923 for the Chinese version (Nie and Jiang 2025) and the original scale's CVI of 0.98 (Chang et al. 2023). This high degree of consistency indicates that experts across different cultural and linguistic contexts universally recognize the core concepts of delirium care self‐efficacy as essential. The expert panels in all three studies—comprising physicians, nurses, and psychiatrists—unanimously agreed on the relevance and appropriateness of the items, leading to the retention of all scale items after minor wording revisions. The minor variations in the reported validity indices among the present study, the original, and the Chinese version can be attributed to methodological and contextual factors. The primary difference in face validity assessment lies in our use of a quantitative Impact Score, which provided an additional, objective metric not explicitly reported in the original study. For content validity, the slight numerical differences in scale‐level CVI values are not substantive; they reflect the inherent variability in ratings from differently composed expert panels, despite their shared expertise. Crucially, all CVI values across studies far exceed the accepted threshold, confirming the construct's universal relevance. The consistent need for minor linguistic revisions across all versions underscores the need for cultural adaptation rather than indicating a weakness, ultimately affirming the scale's robustness and successful cross‐cultural transferability.

The construct validity findings for the P‐DCSE‐I demonstrate a clear, conceptually sound factor structure that aligns closely with the Chinese adaptation (Nie and Jiang 2025) and refines the solution initially proposed in the original study (Chang et al. 2023). Critically, the most significant point of convergence is the confirmation of a stable two‐factor structure across three distinct cultural contexts (Taiwanese, Chinese, and now Iranian), providing robust evidence that this is not an artefact of a single sample but a replicable empirical reality. Our analysis, like that of the Chinese version, successfully extracted two distinct factors—“Confidence in delirium assessment” and “Confidence in delirium management”—which together explained a high cumulative variance (73.6% in our study vs. 65.522% in the Chinese version). This provides strong cross‐cultural evidence that ICU nurses' self‐efficacy in delirium care is a two‐dimensional construct. This finding refines the original study's analysis, which, despite beginning with 26 items and a potential three‐factor solution, ultimately arrived at a stable 13‐item, two‐factor model after removing items with cross‐loadings. Theoretically, this two‐factor structure aligns with Bandura's social cognitive theory (Bandura 1991), which posits that self‐efficacy is task‐specific; the distinction between assessment and management represents two distinct yet related behavioural domains in clinical practice. A key difference lies in the analytical rigour and clarity of the factor solution. Our study employed Maximum Likelihood extraction with Promax rotation, which is considered more appropriate for psychological constructs where factors are expected to be correlated. This method yielded an exceptionally clean solution with all items loading strongly (> 0.715) on their intended factors and no significant cross‐loadings (all below 0.32). In contrast, the original and Chinese studies used Principal Component Analysis (PCA) with Varimax rotation, which assumes uncorrelated factors. The Chinese version reported cross‐loadings on three items (4, 5, 7), which required a decision to assign them to the factor with the higher loading. The absence of such cross‐loadings in our Persian version suggests that the two‐factor structure is particularly stable and well‐defined in our sample.

A major strength of our study is the complementary use of CFA, which was not reported in the original or Chinese validation studies. The strong goodness‐of‐fit indices from our CFA provide strong statistical confirmation that the hypothesized two‐factor model fits the data from Iranian ICU nurses well. This sequential EFA‐CFA approach offers more robust evidence for the scale's structural validity. This methodological advance is a key novel contribution of the Persian validation, as it moves beyond exploratory techniques to confirmatory testing, thereby elevating the psychometric standard of evidence for this scale.

Crucially, our study provides quantitative evidence for convergent and discriminant validity, which was not explicitly reported in the original or Chinese studies (Chang et al. 2023; Nie and Jiang 2025). We established convergent validity, as both CR and AVE values exceeded their respective thresholds of 0.70 and 0.50. This indicates that the items for each factor reliably converge to measure their intended latent construct. Furthermore, discriminant validity was confirmed, as the MSV for each factor was lower than its AVE, demonstrating that the two factors, while related, are statistically distinct and measure different aspects of delirium care self‐efficacy. Hence, the Persian version of the P‐DCSE‐I demonstrates strong construct validity. These additional validity metrics represent a second major novel contribution, as they provide empirical assurance that the subscales are not redundant but capture unique dimensions of the self‐efficacy construct—a critical property for using subscale scores independently in clinical needs assessments. The differences in findings—particularly the cleaner factor solution, the application of CFA, and the formal testing of convergent and discriminant validity—do not indicate discrepancies but rather represent a more comprehensive validation process. These results strengthen the cross‐cultural evidence for the scale's two‐dimensional structure and provide new, robust metrics affirming that it reliably measures two distinct yet related domains of self‐efficacy in delirium care.

In terms of internal consistency, the Cronbach's alpha coefficients for the P‐DCSE‐I were acceptable for both the total scale (*α* = 0.862) and the two subscales (Confidence in delirium assessment: *α* = 0.821; Confidence in delirium management: *α* = 0.847). These values, while slightly lower than the exceptionally high coefficients reported in the original (Total *α* = 0.94; Chang et al. 2023) and Chinese (Total *α* = 0.934; Nie and Jiang 2025) studies, are still considered excellent for psychological instruments and indicate a high degree of inter‐item reliability. The minor variation is likely attributable to sample‐specific characteristics and is not clinically or psychometrically significant, as all values far exceed the acceptable threshold of 0.70. A notable strength of our analysis is the reporting of additional reliability indices, including McDonald's Omega and Maximal Reliability, which confirmed the high internal consistency and provide a more robust assessment than relying solely on Cronbach's alpha.

Regarding test–retest reliability, the results across all three studies are remarkably consistent and indicate excellent stability of the scale over a two‐week interval. The ICC values for the Persian version (Total scale ICC = 0.840) are almost identical to the Pearson correlation coefficients reported for the original scale (Total scale *r* = 0.87; Chang et al. 2023) and the Chinese version (Total scale ICC = 0.954; Nie and Jiang 2025). This high degree of concordance strongly confirms that the P‐DCSE‐I produces stable, reproducible results over time, a critical feature for evaluating interventions.

### 4.1 Limitations

This study had several limitations that should be considered when interpreting the results. First, the sample was recruited exclusively from teaching and medical centers in Ardabil, Iran. This limits the generalizability of the findings to the broader national population of ICU nurses, as cultural and organizational factors may vary in other regions of the country. Moreover, the specific context of Iranian ICU settings—including nurse‐to‐patient ratios, availability of delirium screening protocols, and hierarchical medical structures—may differ substantially from those in Western or East Asian healthcare systems. These contextual factors could influence not only baseline self‐efficacy levels but also the way nurses interpret specific items (e.g., items related to using standardized tools or communicating with physicians). Therefore, the generalizability of the P‐DCSE‐I to non‐Iranian, non‐Middle Eastern, or non‐teaching hospital populations remains untested. Future multi‐center studies across different provinces and healthcare settings are recommended to enhance the external validity of the Persian DCSE‐I. Second, the study relied on self‐reported data, which is susceptible to social desirability bias.

Participants might have overestimated their self‐efficacy levels when responding to the questionnaire. This is particularly relevant in collectivist cultures like Iran, where admitting low confidence may be perceived as a loss of face or professional inadequacy, potentially inflating scores and attenuating true variability. While this is a common limitation in psychometric studies, it should be acknowledged. Future research could incorporate objective performance measures or supervisor ratings to complement self‐reports and mitigate this bias. Third, the test–retest reliability was assessed over a relatively short 2‐week interval. Although this is a standard period, a longer interval would provide stronger evidence for the long‐term stability of the scale scores by better accounting for potential changes in nurses' knowledge or clinical experiences.

Furthermore, the 2‐week interval in our study coincided with routine clinical rotations in some participating units, which may have introduced unmeasured variability due to changes in shift schedules or patient acuity. Fourth, while the sample size was adequate for both EFA and CFA and the participant‐to‐item ratio (16:1) exceeded recommended standards, we conducted the EFA and CFA on the same dataset rather than on two independent subsamples. This was primarily due to the limited and specialized nature of the ICU nursing population in Iran, which constrained our ability to recruit a sufficiently large sample for split‐half validation. Nevertheless, the exceptionally strong factor loadings, excellent fit indices, and theoretical coherence of the extracted factors mitigate the risk of overfitting. However, this same‐dataset approach means that the model's stability should be interpreted with caution until it is cross‐validated in an independent Iranian sample. Future multi‐center studies with larger and more diverse samples are recommended to further confirm the stability of the factor structure through independent cross‐validation.

## 5 Implications for Nursing Practice

Validating the P‐DCSE‐I has several direct and significant implications for nursing practice in Iranian intensive care units. This tool provides nurse managers and clinical educators with a reliable and valid means to objectively assess ICU nurses' perceived self‐efficacy in delirium care. By identifying specific areas of weakness—such as “delirium assessment” or “delirium management”—the scale serves as a needs assessment tool to guide the development of targeted, evidence‐based educational programs and in‐service training. Instead of relying on generic training, educational interventions can be tailored to address the specific competencies where nurses feel less confident. This approach optimizes resource allocation and enhances the effectiveness of continuing education.

Additionally, the P‐DCSE‐I can be essential in evaluating the impact of educational interventions and quality improvement initiatives. By administering the scale before and after a training workshop or the introduction of a new delirium protocol, healthcare institutions can quantitatively assess changes in nurses' confidence levels and provide concrete data on the program's effectiveness. Establishing cutoffs or normative data (once available from larger studies) will further increase its utility for benchmarking and identifying at‐risk units or individuals.

At an individual level, using this scale promotes reflective practice among nurses. Completing the questionnaire encourages them to thoughtfully evaluate their knowledge and skills, heightening their awareness of delirium and motivating self‐directed learning. Ultimately, the regular use of this scale in clinical settings can improve the quality of delirium care, enhance patient outcomes, and support the professional development of ICU nurses in Iran. However, self‐efficacy is a modifiable psychological state rather than a fixed trait; therefore, the scale should be used as a dynamic monitoring tool rather than a one‐time assessment, with repeated measures guiding ongoing professional development efforts.

## 6 Conclusions

The primary aim of this study was to evaluate the psychometric properties of the P‐DCSE‐I for use among ICU nurses in Iran. Given the high prevalence of delirium in critically ill patients and the essential role nurses play in its prevention, recognition, and management, a valid and reliable tool to measure their perceived self‐efficacy is crucial. This study aimed to determine the validity (face, content, and construct) and reliability (internal consistency and test–retest) of the translated scale.

In the settings where this research was conducted—teaching and medical centers—the scale provides nurse managers and clinical educators with a practical tool to identify specific areas where nursing staff lack confidence in delirium assessment and management. This enables the design of targeted, evidence‐based educational programs that address actual competency gaps rather than relying on generic training. Moreover, the scale can be administered before and after educational interventions or protocol implementation to objectively measure effectiveness, guide resource allocation, and support continuous quality improvement efforts at the unit level.

At the broader level of the Iranian healthcare system, the P‐DCSE‐I offers a standardized instrument that can be adopted nationwide to evaluate and benchmark delirium care self‐efficacy across different hospitals, provinces, and ICU settings. The Ministry of Health, nursing regulatory organizations, and hospital accreditation bodies can integrate this scale into their quality monitoring frameworks to identify systemic strengths and weaknesses in delirium care competencies nationwide. This would inform the development of national educational guidelines, support strategic workforce planning, and facilitate the equitable distribution of continuing education resources to regions or institutions with the greatest needs. Furthermore, incorporating the scale into nursing curricula at both undergraduate and postgraduate levels would help assess and enhance students' preparedness for delirium care before they enter clinical practice.

For future applications, the scale can evaluate the impact of large‐scale training programs, conduct cross‐cultural comparisons to understand how different healthcare systems influence delirium care self‐efficacy, and establish national benchmarks through multicenter studies across diverse Iranian provinces. These efforts will ultimately improve the quality of delirium care, enhance patient outcomes, and support the professional development of ICU nurses throughout Iran.

## Author Contributions

**Hossein Fadaei:** conceptualization, investigation, writing – original draft, writing – review and editing, data curation. **Mehraban Shahmari:** conceptualization, methodology, software, writing – review and editing, writing – original draft. **Reza Nemati‐Vakilabad:** conceptualization, visualization, validation, methodology, formal analysis, supervision, project administration, writing – review and editing, writing – original draft, investigation, software. **Fateme Ebrahimi Belil:** conceptualization, methodology, writing – review and editing, writing – original draft, supervision, project administration.

## Funding

The authors have nothing to report.

## Disclosure

The authors used Grammarly for language editing and grammar checking during manuscript preparation. No AI tools were used for data analysis, interpretation, conceptual development, or scientific writing. All scientific content was reviewed, verified, and approved by the authors, who take full responsibility for the manuscript. AI tools were not listed as authors and did not contribute to the intellectual content of the work.

## Ethics Statement

The study proposal received approval from the Ethics Committee of Ardabil University of Medical Sciences (Ethics Code: IR.ARUMS.REC.1403.413, Date of Approval: February 15, 2025). All procedures involving human participants were conducted following the ethical principles outlined in the Declaration of Helsinki. Before their enrollment in the study, written informed consent was obtained from all participating nurses. The consent process ensured that participants were thoroughly informed about the study's objectives, procedures, potential benefits, and any foreseeable risks. They were also assured of the voluntary nature of their participation, their right to withdraw at any time without penalty, and the confidentiality of their data. Participants were informed that their responses would remain anonymous and that no identifying information would be included in any reports or publications. All data were stored securely and accessible only to the research team.

## Consent

The authors have nothing to report.

## Conflicts of Interest

The authors declare no conflicts of interest.

## Data Availability

The data that support the findings of this study are available from the corresponding author upon reasonable request.
